# A Successful Case of TKA With Complex Deformity And Retained Hardware Using Computer Navigation

**DOI:** 10.1016/j.artd.2021.11.005

**Published:** 2021-12-10

**Authors:** Jan Cerny, Jan Soukup, Tomas Novotny

**Affiliations:** Department of Orthopaedics, University J.E. Purkinje, Masaryk Hospital, Usti nad Labem, Czech Republic

**Keywords:** Total knee arthroplasty, Retained hardware, Total knee arthroplasty navigation system, Total knee arthroplasty in special conditions

## Abstract

We present a case report of a 60-year-old Caucasian female patient, who had undergone a series of procedures for a periprosthetic (after total hip arthroplasty) Vancouver C type diaphyseal fracture of the right femur (reverse distal femoral locking compression plate [LCP] osteosynthesis, then a corrective osteotomy with another distal femoral LCP osteosynthesis). Subsequently, she developed high-grade osteoarthrosis of the right knee, indicated for a total knee arthroplasty. Considering the extent of previous procedures, which had significantly compromised the bone quality of the femur and therefore increased the risk of a refracture after an eventual hardware removal, we decided to retain the LCP plate. We concluded that the optimal solution would be the use of a computer-navigated total knee arthroplasty. This procedure obviated the need for intramedullary guiding, while ensuring optimal joint alignment. No postoperative complications emerged.

## Introduction

Total knee arthroplasty (TKA) in patients with retained hardware after previous procedures is still a challenge, even for a highly skilled surgeon. One of the main issues, which is usually faced in such situations, is that the patency of the femoral/tibial canal is often compromised because of the presence of, for example, a nail or a plate. Therefore, utilization of traditional long intramedullary (IM) guidance for component placement is rarely possible.

Alternatives to a long IM guide are several options including use of a short IM or an extramedullar guide. However, these methods usually do not provide optimal precision during implantation. Other approaches, that are not necessarily dependent on IM guidance yet provide excellent precision, include computer navigation, patient-specific instrumentation (PSI), or robotic-assisted surgery.

This article presents a case of a successful TKA in a patient who had undergone a series of procedures on the right femur for a displaced diaphyseal fracture (distal femoral locking compression plate [LCP-DF] osteosynthesis, corrective osteotomy + another LCP-DF), using computer navigation. This technology enabled us to obviate IM guiding and to retain the hardware to avoid the risk of destabilizing the femur, while keeping high-level accuracy in component placement. We hope this case report highlights the possible benefits of computer navigation and inspires other clinicians to use it in such complex cases.

## Case history

**Figure 1:**
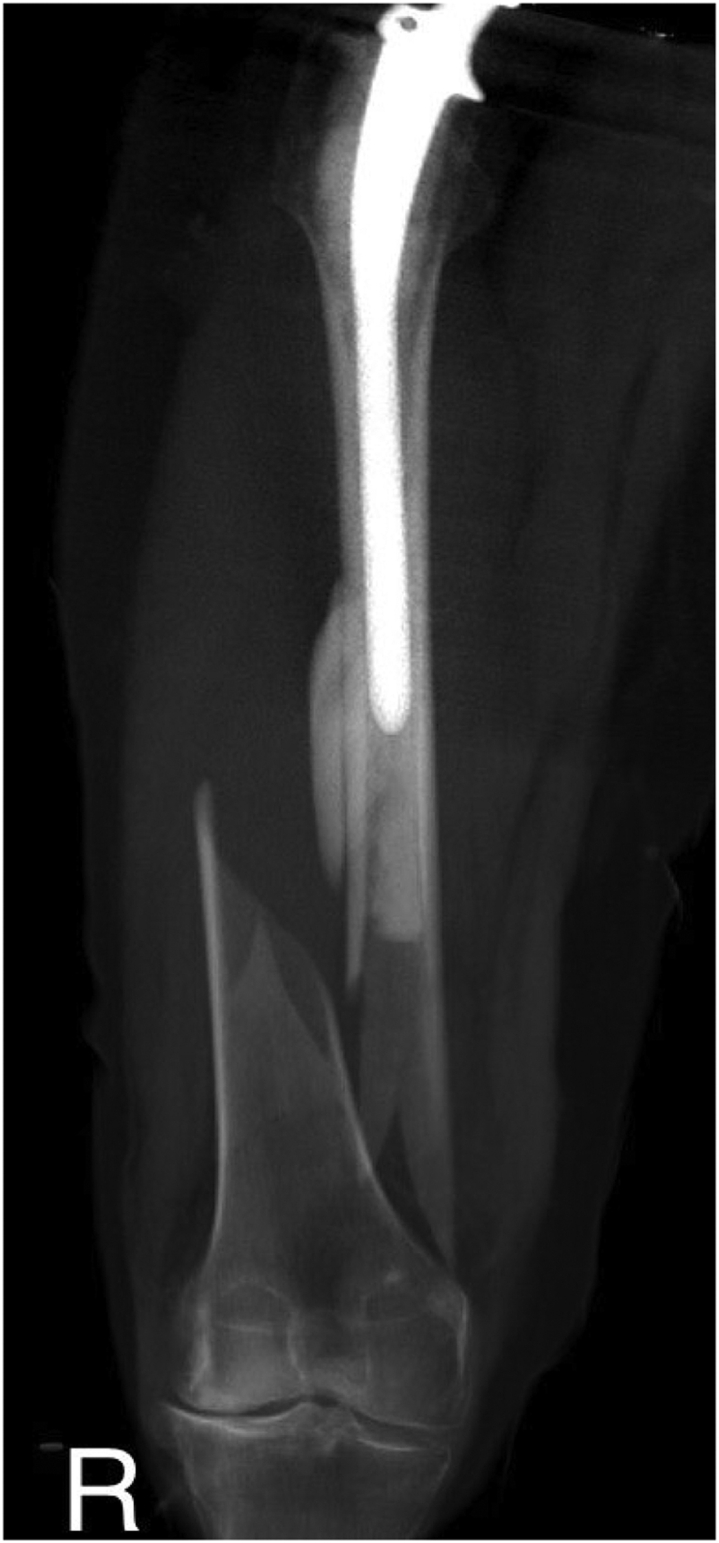
CT scan showing a Vancouver C type diaphyseal fracture of the right femur. The revision hip arthroplasty with a cemented stem remaining intact.

**Figure 2:**
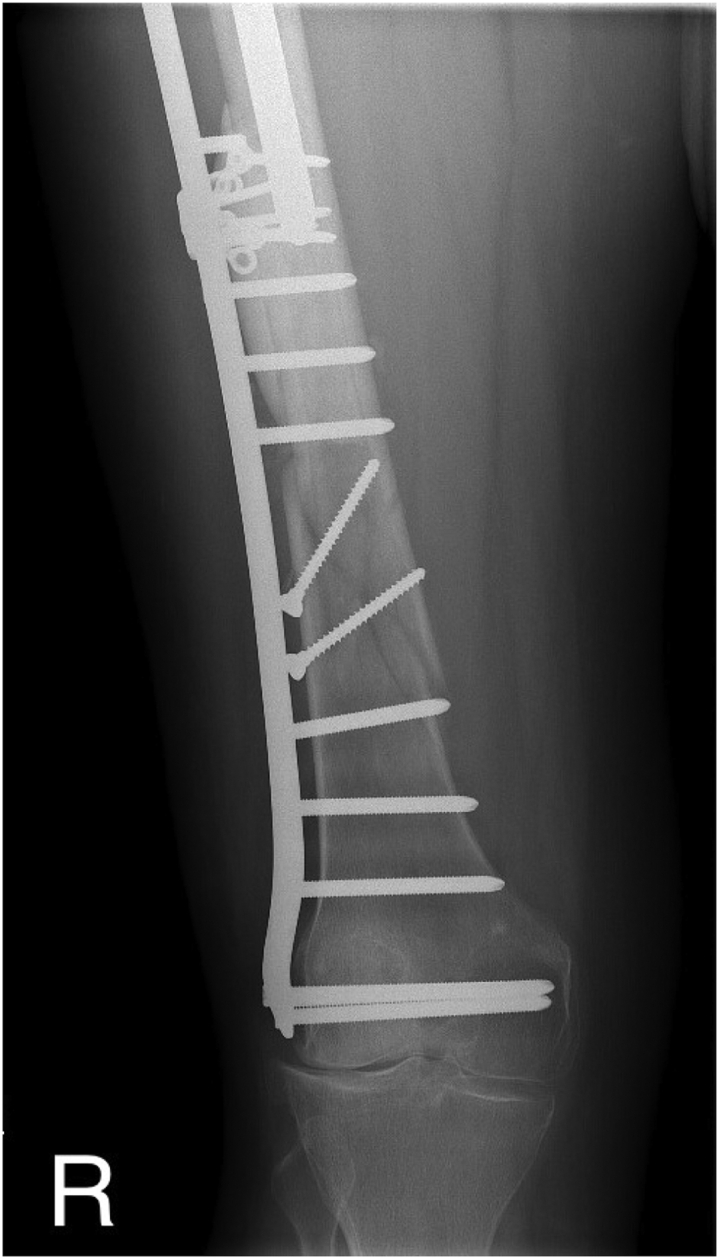
Femoral fracture fixed with an LCP-DF plate with good alignment of the fragments. Slight valgus deviation of the knee with lateral compartment overload is incipient.

**Figure 3:**
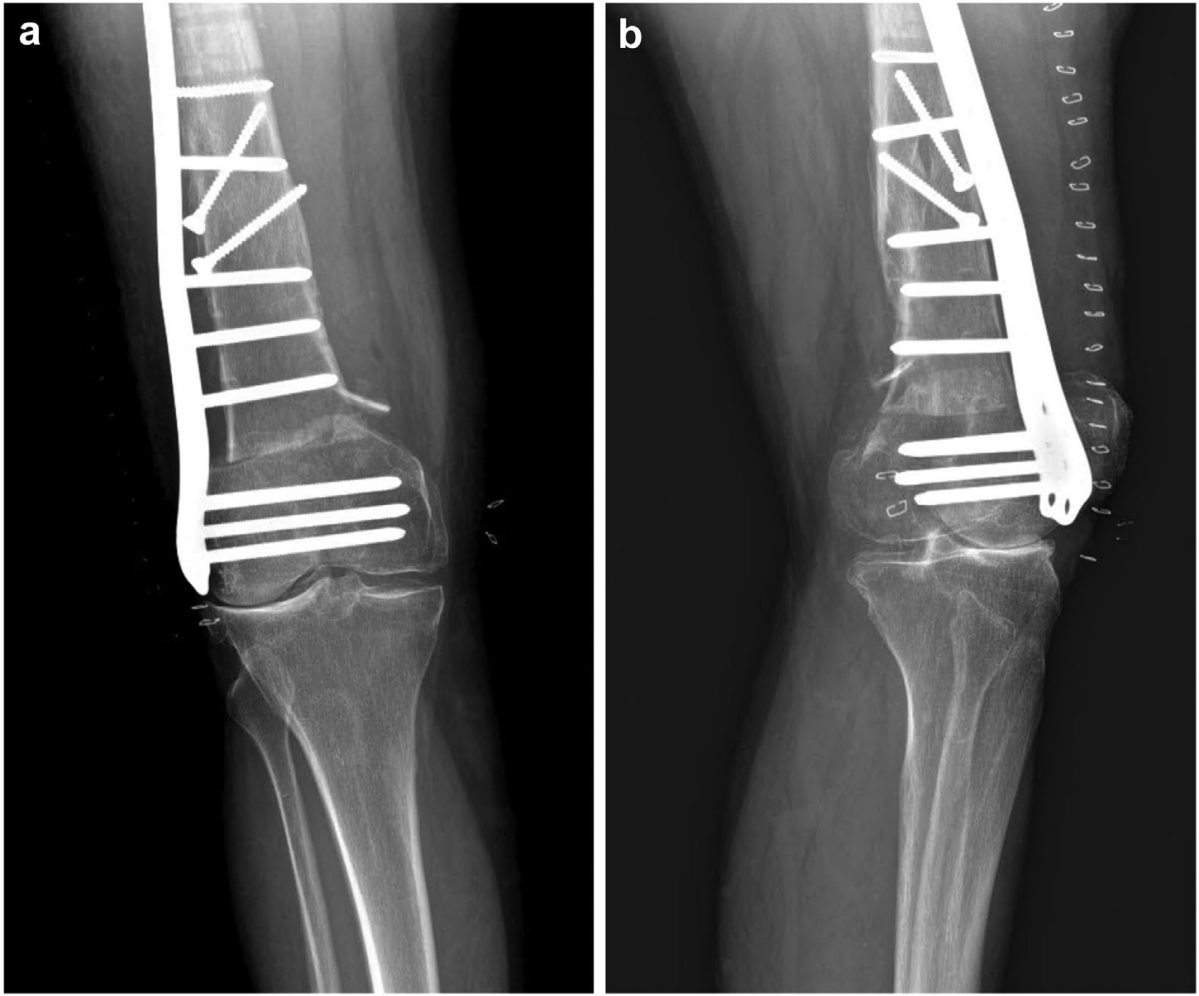
Condition of the femur after the first LCP-DF extraction with a subsequent distal varisation osteotomy, followed by another LCP-DF fixation. A medial cortex osteoporotic compressive fracture of the proximal fragment is visible in the proximity of the osteotomy. (a) Anteroposterior radiograph; (b) TKA detail, lateral radiograph.

Our patient is a 60-year-old Caucasian female with a medical history of arterial hypertension and hyperlipoproteinemia. In 1990, she underwent a total right hip arthroplasty. In 2001, owing to destruction of the acetabulum and severe movement restriction, a revision had to be performed. The dysfunctional cemented cup was exchanged for a noncemented component, fixated with 2 pelvic screws. Furthermore, in 2014, owing to stem loosening and wear of the acetabulum, another revision had to be carried out. This operation included a replacement of the polyethylene acetabular insert, together with an exchange of the stem for a longer cemented component. The second procedure was complicated by the onset of peroneal paresis, which impaired the patient's gait stability. This condition resulted in a displaced periprosthetic Vancouver C type femoral shaft fracture (Fig. 1), after a fall following a stumble in February 2015. This fracture was primarily fixed by LCP-DF osteosynthesis (Fig. 2), providing the patient with a temporary pain-free period. However, after 3 years, a regular follow-up radiograph verified a valgus knee deviation, which accelerated the lateral compartment degeneration. Considering the reoccurrence of pain and knee joint dysfunction, an extraction of the LCP-DF plate, followed by a corrective varisation osteotomy and another LCP-DF fixation (Fig. 3), was recommended. This procedure took place in November 2018. The osteotomy was complicated by a compressive osteoporotic fracture of the medial cortex of the proximal femoral fragment. Fortunately, this fracture did not compromise the stability of the osteosynthesis significantly, as the fragments were pressing against each other. Despite a temporary improvement in the patient’s subjective condition, the typical advanced-stage arthrosis symptoms and radiographic signs continued to progress. After discussion with the patient, it was agreed that we should proceed with a TKA of the affected joint in June 2021.

Because of the history of previous surgeries and biological and iatrogenic complications, which had significantly altered the bone structure of the femur (essentially to the stage of progressive osteoporosis), we decided to retain the hardware in situ as an option of choice. Extracting the plate would probably have put the patient in imminent danger of refracturing the femur, possibly even during postoperative rehabilitation. In these circumstances, we considered a navigated TKA to be a very fitting option, mainly because it provides great precision in component placement without the need for IM guidance [[1], [2], [3]]. Moreover, using the navigation system and leaving the femoral canal intact enabled us to reduce the blood loss [4] and to generally reduce the operation-related trauma. However, in case we needed to remove the plate, we had the extracting hardware ready for use in the operating room. Such situation could have possibly occurred if, after the resection, the layer of intact femoral bone tissue remained thinner than 12 mm. This thickness is generally recommended, but it primarily depends on the type of the TKA [5].

**Figure 4:**
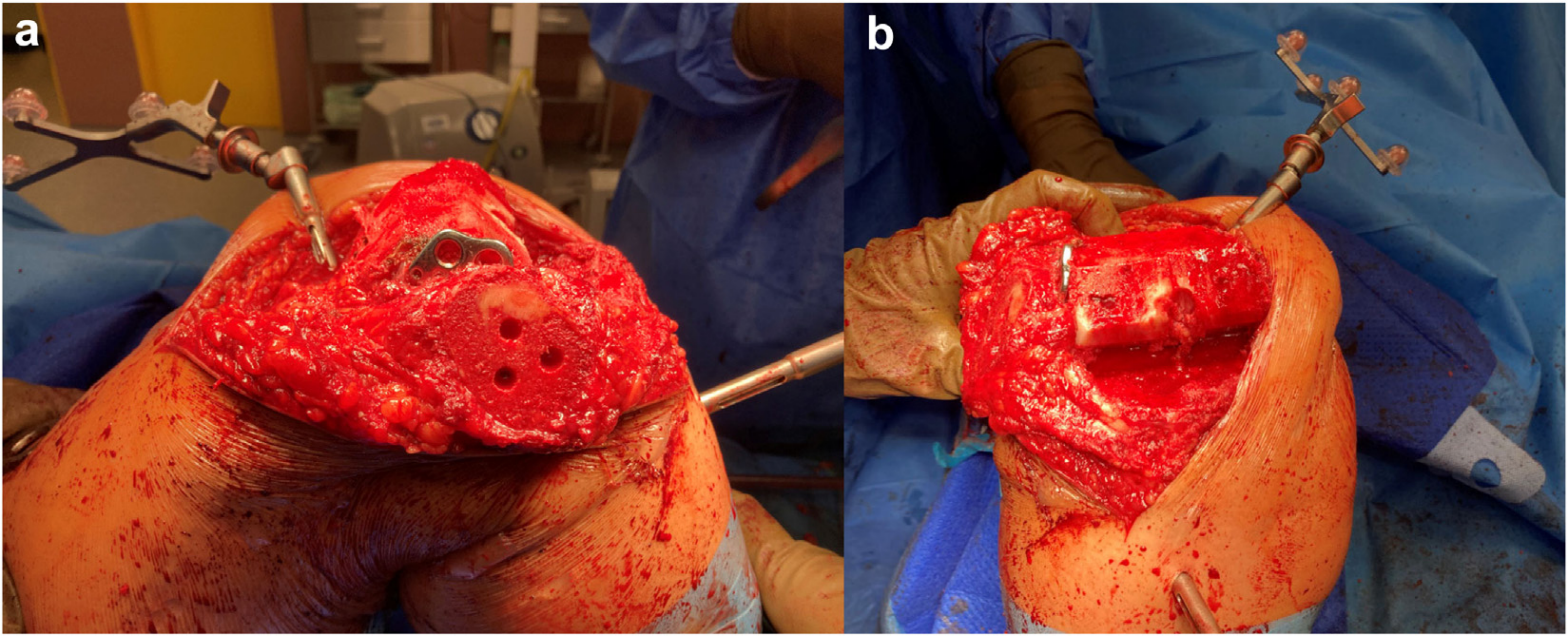
Bone surfaces of the femur after navigation-/template-guided preimplant resection. (a) Lateral view with the visible end of the LCP plate; (b) Frontal view with apparent positioning of the probes.

**Figure 5:**
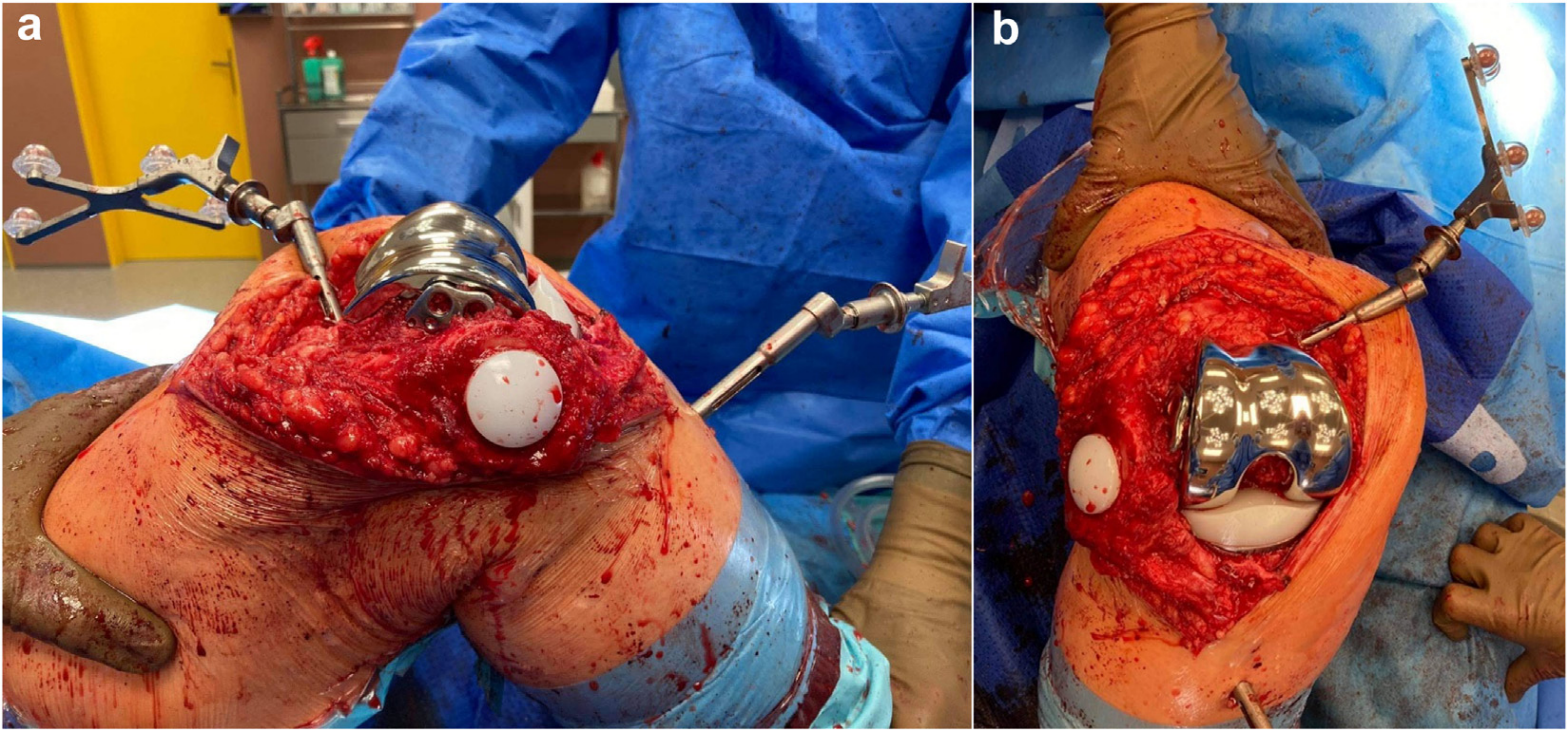
Optimal positioning of the TKA components before suturing. (a) Lateral view; (b) Frontal view.

Our department uses the OrthoPilot Elite knee arthroplasty navigation interface (B. Braun, Aesculap AG, Tuttlingen, Germany). It is an image-free system that uses bicortically fixed transmitter anchorage into the femur and tibia, together with the so-called multitool, which is a handheld device serving as the third probe and intraoperatively as the controller of the system. Basic landmarks that we use throughout the operation are the posterior areas of the femoral condyles, the anterior femoral cortex, the femoral epicondyles, then the intercondylic eminence, followed by the superior tibial articulating planes and finally the 2 malleoli with the center of the ankle. These structures are marked and are submitted to the system with the multitool device. Afterward, using the software, we assess the centers of the hip and the knee, which will help us simulate the preimplant range of motion and any frontal plane (valgus, varus) deformities. These can be evaluated on the basis of the displayed trajectory of the joint movement. The surgeon can determine the order of the bone cuts; in most cases, we start with the tibial resection. In this patient’s case, considering the potential need to extract the plate, we commenced with the femoral cut (Fig. 4a and b). The bone resections can be thoroughly optimized—distal cut height, slope, frontal orientation, and flexion/extension gap. Before the final implantation of the TKA, we checked that the movement of the trial components had no interference with the retained LCP and that the retained hardware had no effect on the level of collateral stability and on the lateral collateral ligament function. Finally, we carried on with cementing the final endoprosthesis (ie, the femoral, tibial, and patellar components) at a distance of 2 mm medially from the retained LCP (Fig. 5a and b). Considering the absence of posterior cruciate ligament insufficiency or severe coronal deformity, we used a cruciate retaining TKA. We finished the procedure by irrigating the wound with a solution of 0.04% polyhexanide. The wound was sutured in a standard manner, and one drain was inserted.

**Figure 6:**
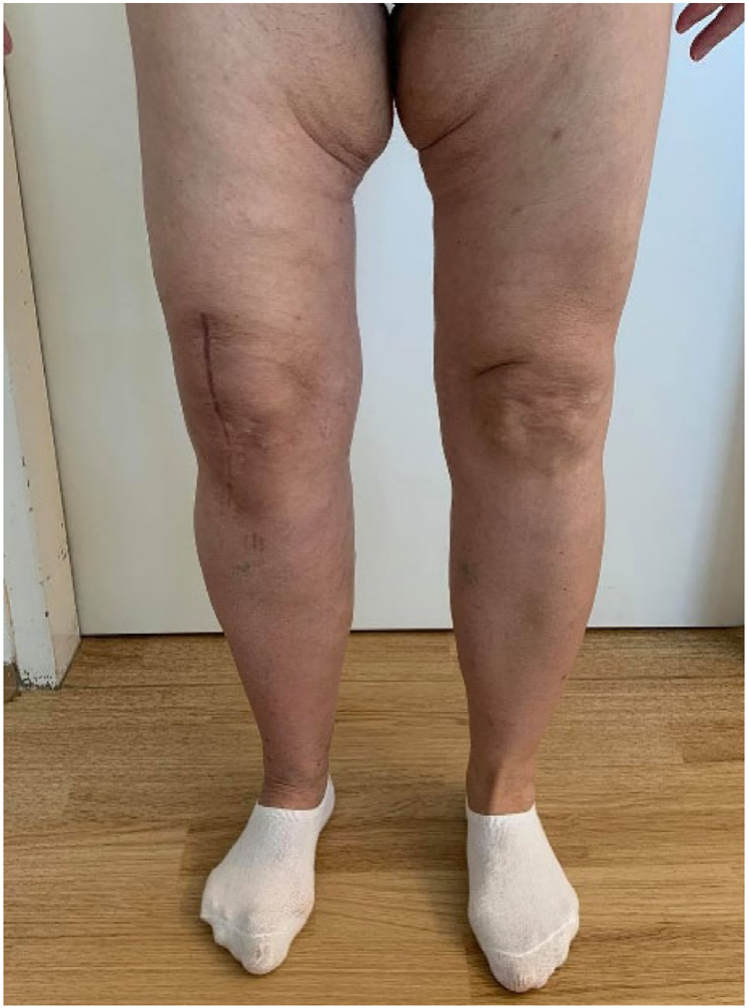
No complications of wound healing of the right knee were encountered. No frontal or sagittal plane knee deformities emerged.

**Figure 7:**
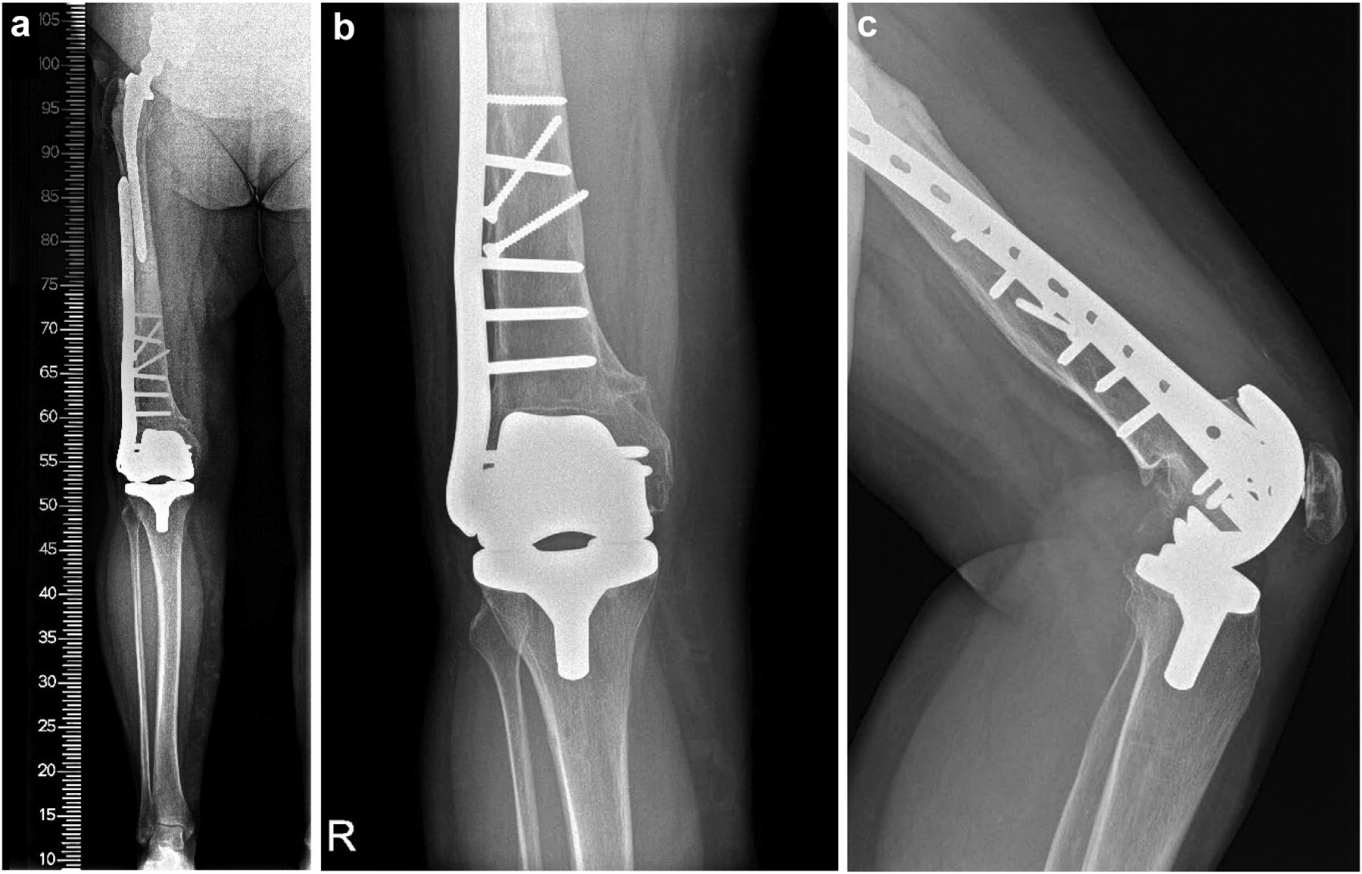
Six-week postoperative radiograph showing optimal alignment of the TKA and no signs of hardware loosening. (a) Large format, including the hip arthroplasty; (b) TKA detail, anteroposterior radiograph; (c) TKA detail, lateral radiograph.

The patient was discharged home on day 7 after the operation, being self-sufficient in ambulation, including stair climbing (using 2 French crutches). The range of motion in the operated joint was 0°-110° in the sagittal plane on the day of discharge, and the motion was painless. We found this result to be more than satisfactory, particularly considering the preoperative 25° extension deficit and the debilitating pain during joint movement. There were no complications in wound healing, as we had extracted the suturing clips on the day when the patient was discharged. The patient was equipped with a standard dose of rivaroxaban, which we routinely prescribe to all post-TKA patients for self-administration until the 14th postoperative day. At the 6-week postoperative checkup, the patient was completely pain-free, with a 0°-125° sagittal range of motion. The patient was still using 2 French crutches, but she was recommended gradually to reduce her dependence on them and to increase the workload of the operated limb. There were no complications in wound healing (Fig. 6). A radiograph showed optimal joint alignment, without any signs of hardware loosening (Fig. 7a-c). At the 6-month postoperative follow-up, the patient was still pain-free, with no further limitations in joint movement, and she was able to walk without aid. Subsequently, a standard follow-up will be done at 1 year postoperatively, and then annually.

## Discussion

Although IM and extramedullary mechanical alignment guides have been greatly improved over the years, there is still 10% global incidence of >3° alignment errors in tibial and femoral component placement in a standard TKA [6], regardless of the surgeon’s experience. If nonstandard anatomical circumstances (such as retained hardware) were also counted in that statistic, the percentage would probably be even higher. There were several authors who also implemented computer navigation in similar cases to ours.

Hamada et al. [2] presented a case of a 59-year-old female who suffered bilateral open fractures of the distal femurs. Subsequently she underwent open reduction internal fixation of both fractures; nevertheless, due to residual malalignment and pain, bilateral TKA was necessary. For the right TKA, they used a CAS system (Stryker Image Enhanced Knee Navigation Ver.2.0; Stryker, Kalamazoo, MI), which is an image-free, extramedullary guidance interface. Six months after the right TKA, the patient underwent a standard, IM guided left TKA, after hardware removal. The postoperative phase was uneventful—the patient has been pain-free, and the range of motion has improved.

Tigani et al. [5] reported on a group of 3 patients who underwent a computer-navigated TKA for posttraumatic arthrosis of the knee, after a prior femoral fracture. Two of the patients underwent a TKA with retained hardware. The orthopedic department tested several navigation systems, including the OrthoPilot, together with the Columbus implant (B. Braun, Aesculap AG, Tuttlingen, Germany). The 2 patients had an uneventful postoperative recovery, with a good clinical outcome.

Manzotti et al. [7] used computer-navigation systems in a group of 16 patients with retained hardware (a distal lateral plate and screws in 7 patients) after a femoral fracture. Ten of the patients were treated with the OrthoPilot system, using the e.motion prosthesis (B. Braun, Aesculap AG, Tuttlingen, Germany). The authors did not observe any navigation system–related complications.

The question of an ideal solution for patients, who could not be treated using IM guidance (due to posttraumatic deformity, retained hardware, history of osteomyelitis, and so on), has been thoroughly addressed by Fehring et al. [8]. Seventeen knees were treated with CAS in this study. The author concluded that CAS is a very beneficial approach, providing more than satisfactory joint alignment, even in otherwise challenging cases.

Our own case presented here is noteworthy in that our patient had already undergone 2 operations before our intervention, and in addition, the corrective osteotomy was complicated with a partial refracture.

Apart from the reasons mentioned previously, there were several other factors that made us incline toward the decision to retain the hardware and to implement a navigated TKA: (1) The navigation system enables us to reduce the extent of the femoral cut. (2) No need for additional incisions to extract the plate. (3) Keeping the plate screws in place means not compromising the postoperative weight-bearing characteristics of the operated limb [3], which is crucial for swift postoperative recovery, especially in older patients with osteoporosis [7].

The typical indication for a post–femoral fracture TKA is posttraumatic arthrosis, which is associated either with a residual malalignment (eg, after osteosynthesis) or with a direct intraarticular injury [9]. In our case, we evaluated the X-ray finding as primary osteoarthritis, but we definitely had to consider the bone damage as a result of the previous injury and procedures. An issue for discussion is the possible need for additional extra-articular (or intra-articular) corrective osteotomies, to obtain proper alignment. Several studies [4,7] have proved that, thanks to the capability of intraoperative mechanical axis assessment (when using navigation systems), we can avoid such osteotomies more frequently than when standard TKA is used.

The use of PSI is another possible approach in cases of retained hardware and/or extraarticular deformity [10]. While being an interesting option, our department does not yet have experience with this technology.

Robotic-assisted surgery is another option, which can possibly exceed the capabilities of CAS (eg, in terms of accuracy), yet again, such technology is not available in our department.

We concluded that especially the distal portion of the plate was crucial for femoral stability; therefore, from our point of view, even a partial excision of the hardware (through metal cutting) would not be an ideal solution.

However, retaining hardware in all knees just because of the possibility to use modern systems (navigation, PSI, robotics, and so on) cannot be advocated. If we had a patient with ambulation-related pain in the affected area, for example, due to a prominent plate, extraction would naturally be favorable. Another topic for discussion is the longer operation time when using navigation than when using standard TKA. This prolongation is mainly caused by the need to install the probes and due to the continuous control of their positioning throughout the procedure. We anticipate that, with routine utilization of navigation systems, the duration of the surgery will be shortened.

## Summary

The use of computer-assisted navigation systems in patients with an extraarticular deformity and/or retained hardware after prior procedures is a beneficial alternative to a standard TKA. We are convinced that, in the case of our patient, the use of such systems enabled us to lower the potential risk of a refracture of the femur due to its notable weakening after earlier procedures. The main benefit is the possibility to avoid IM guiding for the implantation of particular components, while still retaining more than sufficient precision in their placement. Furthermore, by keeping the hardware in place, patients can avoid the need for a two-stage procedure, which is associated with overall greater operation trauma and 2 separate anesthesias. However, we must evaluate the patient comprehensively, considering all the circumstances (age, physical shape, subjective state before the TKA, and so on), to provide individually optimized treatment.

## Funding

The study was supported by the Krajska zdravotni a.s., Usti nad Labem, 10.13039/100014809Czech Republic (grant no.: IGA-KZ-217116003).

## Conflicts of interest

The authors declare that they have no known competing financial interests or personal relationships that could have appeared to influence the work reported in this article.

The authors declare the following financial interests/personal relationships which may be considered as potential competing interests:

## Informed patient consent

The authors confirm that informed consent has been obtained from the involved patients or if appropriate from the parent, guardian, power of attorney of the involved patients; and, they have given approval for this information to be published in this case report (series).

## Appendix A Supplementary data

Conflict of Interest Statement for Cerny

Conflict of Interest Statement for Novotny

Conflict of Interest Statement for Soukup
