# Supplementary material for: A Successful Case of TKA With Complex Deformity And Retained Hardware Using Computer Navigation
Source: Arthroplast Today. 2021 Dec 10;13:29–34. doi: 10.1016/j.artd.2021.11.005 (PMC8666612; doi:10.1016/j.artd.2021.11.005)
Supplement: Conflict of Interest Statement for Novotny [file mmc2.pdf]

CONFLICT OF INTEREST STATEMENT

American Association of Hip and Knee Surgeons

(Adopted from the American Academy of Orthopaedic Surgeons disclosure statement)

The following form **must be filled out completely and submitted by each author (example, 6 authors, 6 forms). All items require a response. If there is no relevant disclosure for a given item, enter "*None*."**

Computer–Navigated Total Knee Arthroplasty as the only Safe Solution for a Patient with a Retained LCP–DF after a Femoral Fracture.

1. Royalties from a company or supplier (The following conflicts were disclosed)

   None

2. Speakers bureau/paid presentations for a company or supplier (The following conflicts were disclosed)

   None

3A. Paid employee for a company or supplier (The following conflicts were disclosed)

   None

3B. Paid consultant for a company or supplier (The following conflicts were disclosed)

   None

3C. Unpaid consultants for a company or supplier (The following conflicts were disclosed)

   None

4. Stock or stock options in a company or supplier (The following conflicts were disclosed)

   None

5. Research support from a company or supplier as a Principal Investigator (The following conflicts were disclosed)

   None

6. Other financial or material support from a company or supplier (The following conflicts were disclosed)

   None

7. Royalties, financial or material support from publishers (The following conflicts were disclosed)

   None

8. Medical/Orthopaedic publications editorial/governing board (The following conflicts were disclosed)

   None

9. Board member/committee appointments for a society (The following conflicts were disclosed)

   None

Each author must sign AND print or type his/her name, date and submit a separate form

In addition, one BLINDED Conflict of Interest form (no author names used) should be submitted per manuscript with all author disclosures.

Tomas Novotny, MD, Ph.D.		09/08/2021
Author Name (Print or Type)	Author Signature	Date
